# Brief Vigorous Stair Climbing Effectively Improves Cardiorespiratory Fitness in Patients With Coronary Artery Disease: A Randomized Trial

**DOI:** 10.3389/fspor.2021.630912

**Published:** 2021-02-16

**Authors:** Emily C. Dunford, Sydney E. Valentino, Jonathan Dubberley, Sara Y. Oikawa, Chris McGlory, Eva Lonn, Mary E. Jung, Martin J. Gibala, Stuart M. Phillips, Maureen J. MacDonald

**Affiliations:** ^1^Department of Kinesiology, McMaster University, Hamilton, ON, Canada; ^2^Hamilton Health Sciences, ON, Canada; ^3^School of Kinesiology and Health Studies, Queens University, Kingston, ON, Canada; ^4^Population Health Research Institute, Hamilton, ON, Canada; ^5^Department of Medicine, McMaster University, Hamilton, ON, Canada; ^6^School of Health and Exercise Sciences, University of British Columbia, Kelowna, BC, Canada

**Keywords:** high intensity interval training, cardiac rehabilitation, exercise, coronary artery disease, cardiorespiratory fitness

## Abstract

**Background:** Cardiac rehabilitation exercise reduces the risk of secondary cardiovascular disease. Interval training is a time-efficient alternative to traditional cardiac rehabilitation exercise and stair climbing is an accessible means. We aimed to assess the effectiveness of a high-intensity interval stair climbing intervention on improving cardiorespiratory fitness ($\dot{\text{V}}{\text{O}}_{2\text{peak}}$) compared to standard cardiac rehabilitation care.

**Methods:** Twenty participants with coronary artery disease (61 ± 7 years, 18 males, two females) were randomly assigned to either traditional moderate-intensity exercise (TRAD) or high-intensity interval stair climbing (STAIR). $\dot{\text{V}}{\text{O}}_{2\text{peak}}$ was assessed at baseline, following 4 weeks of six supervised exercise sessions and after 8 weeks of ~24 unsupervised exercise sessions. TRAD involved a minimum of 30 min at 60–80%HR_peak_, and STAIR consisted of three bouts of six flights of 12 stairs at a self-selected vigorous intensity (~90 s/bout) separated by recovery periods of walking (~90 s). This study was registered as a clinical trial at clinicaltrials.gov (NCT03235674).

**Results:** Two participants could not complete the trial due to the time commitment of the testing visits, leaving *n* = 9 in each group who completed the interventions without any adverse events. $\dot{\text{V}}{\text{O}}_{2\text{peak}}$ increased after supervised and unsupervised training in comparison to baseline for both TRAD [baseline: 22.9 ± 2.5, 4 weeks (supervised): 25.3 ± 4.4, and 12 weeks (unsupervised): 26.5 ± 4.8 mL/kg/min] and STAIR [baseline: 21.4 ± 4.5, 4 weeks (supervised): 23.4 ± 5.6, and 12 weeks (unsupervised): 25 ± 6.2 mL/kg/min; *p* (time) = 0.03]. During the first 4 weeks of training (supervised) the STAIR vs. TRAD group had a higher %HR_peak_ (101 ± 1 vs. 89 ± 1%; *p* ≤ 0.001), across a shorter total exercise time (7.1 ± 0.1 vs. 36.7 ± 1.1 min; *p* = 0.009). During the subsequent 8 weeks of unsupervised training, %HR_peak_ was not different (87 ± 8 vs. 96 ± 8%; *p* = 0.055, mean ± SD) between groups, however, the STAIR group continued to exercise for less time per session (10.0 ± 3.2 vs. 24.2 ± 17.0 min; *p* = 0.036).

**Conclusions:** Both brief, vigorous stair climbing, and traditional moderate-intensity exercise are effective in increasing $\dot{\text{V}}{\text{O}}_{2\text{peak}}$, in cardiac rehabilitation exercise programmes.

## Introduction

In Canada, cardiovascular diseases are the second leading cause of death in both men and women (Lavie et al., 2009). A cornerstone for prevention of secondary cardiovascular disease following percutaneous cardiac procedures is cardiac rehabilitation and lifestyle change; however, fewer than 50% of referred patients engage in cardiac rehabilitation despite a substantial reduction in risk for a secondary event (Grace et al., 2016). There has been an increasing focus on “alternative” forms of cardiac rehabilitation (Clark et al., 2015; Anderson et al., 2017) to meet patient preference and address barriers to participation and adherence. Patients undertaking cardiac rehabilitation have, as an example, expressed interest in having more home-based programmes (Clark et al., 2015). The focus on shorter, more intense, and yet effective, exercise training may be an ideal option. Provision of alternative forms of aerobic exercise in cardiac rehabilitation would offer choice and may increase program uptake and maintenance. A growing body of evidence demonstrates that high-intensity interval training (HIIT) can be an effective and safe alternative to traditional moderate-intensity continuous training (TRAD) (Quindry et al., 2019), by inducing similar, or even superior, physiological adaptations in both healthy and diseased populations (Adams et al., 2008; Guiraud et al., 2012; Rognmo et al., 2012; Currie et al., 2013a; Aamot et al., 2014; Hannan et al., 2018). Furthermore, engagement in vigorous exercise was associated with lower all-cause mortality in a study recently completed in ~15,000 patients with stable coronary artery disease (CAD) from 39 countries (Stewart et al., 2017).

To date, the majority of HIIT interventions have been conducted in laboratory- or hospital-based settings where exercise is completed on either a treadmill or stationary bicycle ergometer (Currie et al., 2013a; Conraads et al., 2015; Villelabeitia Jaureguizar et al., 2016; Keech et al., 2020). In some previous studies, when compared to TRAD, enhancements in maximal cardiorespiratory fitness and measures of cardiovascular function with HIIT were equivalent despite a dramatically reduced time commitment. When exercise training duration was not different and >7 weeks in length, a meta-analysis on 17 studies reported HIIT improved cardiorespiratory fitness significantly more compared to TRAD (Hannan et al., 2018). Stair climbing has been suggested to be a feasible alternative to TRAD (Halsey et al., 2012; Caruthers et al., 2018) and stair climbing interventions have been shown to be efficacious in inducing improvements in cardiorespiratory fitness, cardiovascular health, strength, and balance in older persons (Donath et al., 2014; Webb et al., 2016). Recent work has shown that brief, vigorous stair climbing elicits improvements in cardiorespiratory fitness in young, sedentary adults, comparable to stationary cycling-based HIIT (Allison et al., 2017), and was well-tolerated in those with Type 2 Diabetes (Godkin et al., 2018).

In cardiac rehabilitation, the addition of alternative programmes, such as home-based services, seems to increase adherence (Grace et al., 2014; Thomas et al., 2019), while still resulting in similar improvements in cardiorespiratory fitness, quality of life, and cardiovascular disease risk factor management compared to supervised cardiac rehabilitation programmes (Aamot et al., 2014; Anderson et al., 2017). Further, the combination of both supervised (center- or hospital-based) and unsupervised (home-based) cardiac rehabilitation programmes may be a more compelling and effective model (Bravo-Escobar et al., 2017; Gabelhouse et al., 2018).

The purpose of this study was to examine the effectiveness of a stair climbing-based HIIT protocol on improving peak oxygen uptake ($\dot{\text{V}}{\text{O}}_{2\text{peak}}$) in patients with CAD completing outpatient cardiac rehabilitation. The program consisted of 6 supervised exercise sessions over 4 weeks, followed by 8 weeks of unsupervised exercise (~24 exercise sessions) during a 12-week intervention. We compared the effects of vigorous stair climbing (STAIR) to standard cardiac rehabilitation programmes (TRAD) on changes in $\dot{\text{V}}{\text{O}}_{2\text{peak}}$, program adherence, quality of life, and enjoyment. We hypothesized that 12 weeks of both STAIR and TRAD would improve cardiorespiratory fitness and quality of life in patients with CAD enrolled in cardiac rehabilitation exercise training.

## Methods

### Participants

Patients with CAD were recruited from the Cardiac Health and Rehabilitation Center (CHRC) in the Hamilton General Hospital. To be eligible, participants needed to be registered for cardiac rehabilitation and have had a history of myocardial infarction, coronary artery bypass graft and/or percutaneous coronary intervention. Exclusion criteria consisted of previous participation in rehabilitation for the same cardiac event, having had a non-cardiac surgical procedure ≤ 2 months prior to recruitment, a pacemaker or atrial fibrillation, documented peak orifice area valve stenosis, symptomatic peripheral arterial disease that limits exercise capacity, unstable angina, uncontrolled hypertension (blood pressure >180/100 mmHg), documented chronic obstructive pulmonary disease (FEV_1_ <60% and/or FVC <60%) and any musculoskeletal abnormality that would limit exercise participation. This study was approved by the Hamilton Integrated Research Ethics Board (#3301) and conformed to the Declaration of Helsinki. This study was registered as a clinical trial at clinicaltrials.gov (NCT03235674). As reported on the clinical trial registry, the primary outcome of this non-inferiority, repeated measures, randomized trial is brachial artery flow mediated dilation and that variable will be reported in a separate manuscript along with more detailed cardiovascular physiology measures. This study addresses the overall trial design and presents the cardiorespiratory fitness, measured by $\dot{\text{V}}{\text{O}}_{2\text{peak}}$, as the primary outcome of this paper.

### Study Design

**Figure 1:**
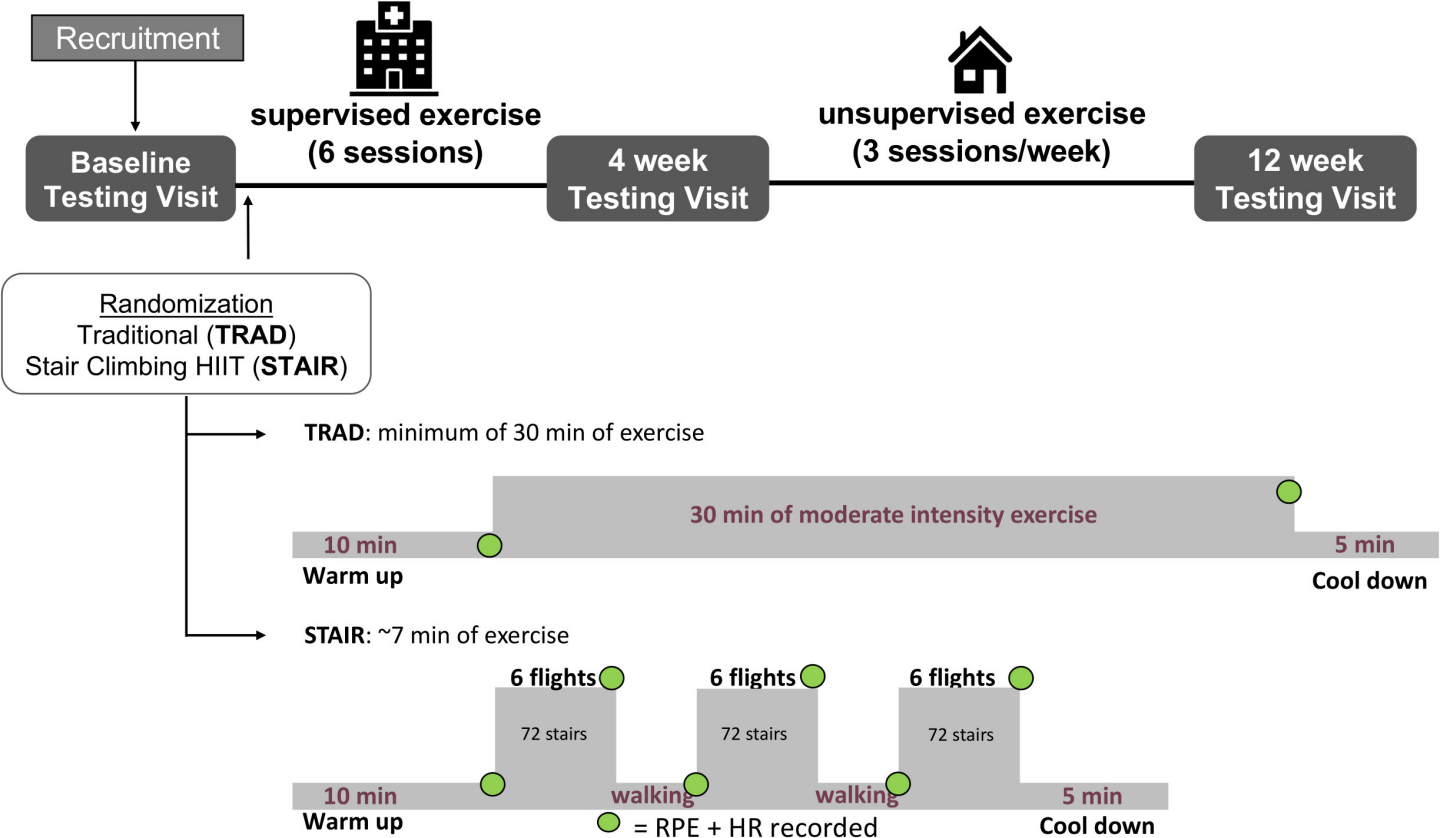
Illustration of the study timeline and exercise interventions. Participants were recruited and completed the baseline testing visit and then were randomized to either the traditional moderate-intensity continuous training (TRAD) or stair climbing high intensity interval training (STAIR) groups. The participants completed six sessions of supervised exercise at the Cardiac Health and Rehabilitation Center (CHRC) and then underwent the second testing visit. Thereafter, the participants completed unsupervised exercise for an additional 8 weeks and then completed the final testing visit. TRAD consisted of a minimum of 30 min of moderate intensity exercise at 60–80% peak heart rate. STAIR consisted of three bouts of climbing a single flight of stairs 6 times at a self-selected, but vigorous pace (14–15/20 RPE) separated by a ~90 s periods of active recovery at a comfortable pace (~7 min total of high and low intensity intervals). For both exercise protocols heart rate was monitored continuously throughout the exercise session. Each of the estimated exercise times include the 10 min warm-up and 5 min cooldown. RPE and HR were recorded on paper at the specific timepoints indicated by a green dot. RPE, rating of perceived exertion; HR, heart rate. The hospital icon was created by Nibras@design, the nounproject.com. All other icons were developed using Microsoft PowerPoint (version 16.45 for Mac).

Participants enrolled in the study underwent baseline screening assessments as depicted in Figure 1, including a clinically supervised cardiopulmonary exercise test (CPET) test to determine $\dot{\text{V}}{\text{O}}_{2\text{peak}}$. All prescribed medications and vitamins were taken as usual before the CPET. Prior to exercise program initiation, participants were randomly allocated into either the traditional moderate-intensity continuous (TRAD) or stair-climbing-based HIIT (STAIR) groups. Prior to exercise group allocation, the randomization scheme was generated by one study investigator using the Web site Randomization.com (http://www.randomization.com). A second study investigator enrolled participants and the group allocation of the respective participant was revealed after consent. Three testing visits were completed at McMaster University over the course of the 12 weeks of the study: before training (baseline), following 6 supervised sessions (~4 weeks) and after 8 additional weeks of ~24 unsupervised exercise sessions (total of 12 weeks). All tests were scheduled to take place at the same time of day in the morning. For these tests, participants were asked to arrive at the laboratory in a fasted state, having abstained from exercise and alcohol consumption for 24 h and caffeine consumption for 10 h. All prescribed medications and vitamins were taken as usual, except for any vasoactive medications (i.e., nitroglycerin). The testing included the following physiological assessments measurements of resting heart rate, blood pressure, height, body mass, and BMI. The quality of life questionnaire was administered prior to a venous blood draw. Habitual activity information was collected at the end of the visit via self-reported questionnaire and accelerometer data.

### Measurements

#### Cardiopulmonary Exercise Stress Test (CPET)

Symptom-limited incremental CPETs were completed under medical supervision, at the Hamilton General Hospital, using either an electronically braked cycle ergometer or a treadmill, with direct gas monitoring to assess $\dot{\text{V}}{\text{O}}_{2\text{peak}}$ using a metabolic cart (SensorMedics Vmax 229; California, USA) as an indicator of cardiorespiratory fitness. During the tests, participants were monitored each minute for blood pressure via manual measurement using a stethoscope and aneroid sphygmomanometer (Durashock DS66, Welch Allyn, New York, USA) and monitored continuously for heart rate using a 12-lead electrocardiogram. The cycle workload was increased by 100 KPM per minute (16.34 W). Treadmill workload began at 3.2 km/h and 0% grade and after the first minute the speed increased to 4.8 km/h. Thereafter, the incline increased by 2.5% per minute. Once the grade reached 20%, the speed was increased by 0.8 km/h each minute.

#### Anthropometric and Blood Measures

Anthropometric measurement of height (m) was obtained at the first testing visit while body mass (in kg) was obtained at each visit and used to determine body mass index (in kg/m^2^). Plasma samples for fasting glucose, lipid and insulin concentrations were obtained at each testing visit. Resting blood pressure was assessed in triplicate after a 10 min supine rest (Dinamap V100, GE Healthcare, Chicago, IL). If the systolic blood pressure measurements differed by >5 mmHg, a fourth measurement was taken, and a minimum of three measurements were averaged. Standard venipuncture techniques were used to collect two 4-mL blood samples (BD Vacutainer Plus, Red BD Hemogard Closure, Franklin Lakes, NJ) at the first testing visit. Venous blood samples were spun at 4,000 rpm at 4°C for 10 min (Sorvall Legend XTR Centrifuge, Thermo Scientific, Waltham, MA). Serum was aliquoted into three polypropylene tubes (Falcon, Corning Science, Corning, NY) and frozen at −20°C. Frozen samples were sent to the Hamilton Regional Medicine Program Core Laboratory for analysis of fasted glucose and insulin, HDL, LDL, triglycerides, cholesterol, non-HDL cholesterol which were used to calculate the total cholesterol:HDL ratio by dividing total cholesterol number by HDL cholesterol number. The assays for blood analysis are as follows. Plasma glucose concentrations were measured in triplicate using colorimetry (Vitros XT 7600, Ortho Clinical Diagnostics, New Jersey, USA). Plasma insulin concentrations were measured in triplicate using the immunometric method (Vitros XT 7600, Ortho Clinical Diagnostics, New Jersey, USA). Cholesterol (total, high-density lipoprotein [HDL], and low-density lipoprotein [LDL]) and triglycerides (TG) were analyzed in triplicate using colorimetry (Vitros XT 7600, Ortho Clinical Diagnostics, New Jersey, USA). Intra- and inter-assay CVs for all techniques were <5 and <6%, respectively.

#### Habitual Physical Activity

Both objective and subjective measures of habitual physical activity were assessed at all three experimental timepoints. Objective habitual physical activity was assessed using an ActiGraph™ wrist-worn accelerometer for 7 days prior to each testing timepoint to estimate daily energy expenditure, step-count, physical activity amount and intensity. Energy expenditure was estimated via conversion of tri-axial counts, vector magnitude, and anthropometric measurements using the Freedson et al. (1998). Subjective habitual physical activity was also assessed using the 7-day recall IPAQ.

#### Quality of Life

Quality of life was assessed via self-report using the MacNew Quality of Life After Myocardial Infarction (QLMI). This validated, interviewer-administered, condition-specific health-related quality of life questionnaire (Höfer et al., 2004) was completed at baseline, 4, and 12 weeks of training. This questionnaire consists of 27 items which fall into three domains: emotional function domain (14 items), physical function domain (13 items), and social function domain (13 items). Each question is answered on a 7-point scale where 7 is high health-related quality of life and 1 is poor health-related quality of life. The maximum score indicating the highest quality of life is 175 arbitrary units.

#### Exercise Enjoyment

Perceived enjoyment, one indicator of likelihood to engage in a specific type of exercise in the future (Kendzierski and DeCarlo, 1991), was measured twice; after the completion of both the supervised portion and unsupervised exercise training using the Physical Activity Enjoyment Scale (PACES). This 18-item scale consists of a series of questions that are answered using a 7-point bipolar Likert scale. After the questionnaire was completed, the scoring was calculated where reversed negative items are converted to positive values, as per the guidelines (Kendzierski and DeCarlo, 1991). Two of the questions are reverse-coded, if a low score indicated a higher exercise enjoyment, then it was converted to a positive value, for example, with a score of 1 being converted to a 7 in the calculation. The minimum and maximum possible scores are 18 and 126 arbitrary units, which correspond to a lowest and highest exercise enjoyment, respectively.

#### Exercise Training

In accordance with current program practice at the CHRC, the first six training sessions (TRAD and STAIR) were conducted in the CHRC and supervised by registered kinesiologists in an individualized setting. Once the first six sessions were completed, patients were referred to a community-based exercise and rehabilitation program or given the option to exercise at home. In this study, every exercise session involved a 10 min warm up and a 5 min cool-down consisting of light walking. A description of the study design and both training programs are presented in Figure 1. Each participant was provided with a receiver (watch) and a corresponding heart rate sensor chest strap (Model A300, Polar H9 heart rate sensor, Polar Electro Oy, Finland). The data from each exercise session was subsequently downloaded using software available online (Polar FlowSync 3.0.0, Polar Electro 2018). Once participants completed six exercise training sessions (TRAD or STAIR) at the CHRC, a second CPET was administered. Participants were then instructed to continue their prescribed exercise program independently, aiming for three exercise sessions per week. Each participant was asked to use the portable heart rate monitor to record every exercise session in addition to recording their sessions in an exercise logbook. Exercise adherence for the unsupervised exercise training period (weeks 5–12) was calculated where a total of 24 sessions was considered 100% adherence, which was determined by the prescription of three sessions per week for 8 weeks.

#### Traditional Moderate-Intensity Continuous Training (TRAD)

The exercise intensity was individualized for the TRAD group using a target training heart rate determined from individual CPET results using the heart rate-reserve (HRR) method. The TRAD exercise program included a combination of stationary cycling, treadmill and/or self-paced walking. All participants in the TRAD group were advised to accumulate a minimum of 30 min of exercise per session in addition to the 10 min warm-up and 5 min cool-down periods and were not restricted from exercising longer if they desired to do so. Each exercise session, regardless of modalities involved, was performed at a workload designed to elicit 60–80% of the individual HRR determined from the pre-training CPET and with an intensity goal of 11–13 on Borg's Rate of Perceived Exertion (RPE) 6–20 scale (Borg et al., 1987). The percentage of heart rate-reserve (%HRR) was calculated after the exercise intervention was complete.

#### Stair Climbing-Based High Intensity Interval Training (STAIR)

The STAIR protocol was modeled after previous work (Allison et al., 2017) that showed stair climbing was effective for improving cardiorespiratory fitness in sedentary adults. The protocol consisted of a 10 min warm-up and 5 min cool-down of self-paced walking on flat ground, and three exercise bouts that each involved continuously ascending and descending a single flight of stairs six times (12 steps). Each of the three stair climbing bouts was separated by a 90-s period of active recovery. For the stair climbing bouts, participants were instructed to, “Climb up and down the stairs one step at a time. Ascend at a pace that you find challenging, and descend at a pace you find comfortable, such that you feel you can safely manage the three bouts of stair climbing. Use the railings for support if you wish.” For the recovery bouts, participants were instructed to walk at a self-selected pace on flat ground. Participants were asked to aim for an RPE of 14–15/20, and were asked to identify their overall effort and fatigue immediately following the completion of each bout by providing an overall RPE (Borg et al., 1987) that they felt corresponded to the entirety of the previous high intensity bout (i.e., ascent and descent). For the purpose of exercise intensity comparison with the TRAD group %HRR was calculated and was not used for exercise prescription of the STAIR group.

### Statistics

All statistical analyses were performed using Statistical Package for Social Science software (version 23; SPSS Inc., Chicago, IL). Independent Student's *t-tests* were used to assess differences in supervised and unsupervised exercise protocol outcomes between exercise groups. All interventional outcome data was compared using a two-way repeated measures ANOVA with two levels of group (TRAD and STAIR) and three levels of time (baseline, 4 weeks, and 12 weeks). A Tukey's HSD *post-hoc* test was used to assess interaction effects. Data are presented as mean ± SD, unless otherwise noted, and for all analyses, the level of alpha was set at 0.05. An a priori power calculation was completed on the primary outcome, endothelial function, as reported in the clinical trial registry. In this paper, however, we are reporting on several of the secondary outcome measures and specifically cardiorespiratory fitness as an important indicator of effectiveness of exercise training programs.

## Results

**Figure 2:**
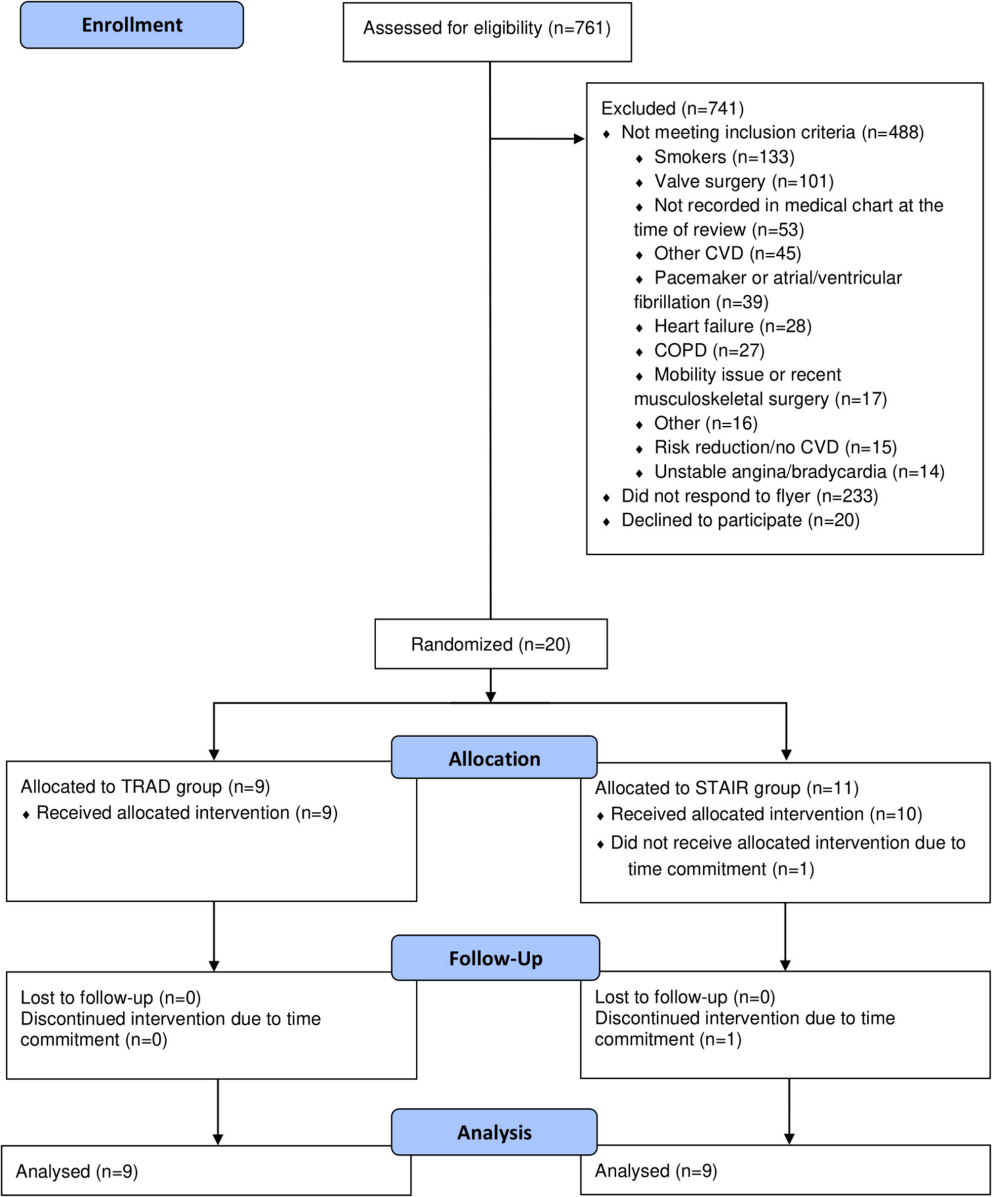
CONSORT flow diagram. CONSORT, Consolidated Standards of Reporting Trials; TRAD, Traditional moderate-intensity continuous exercise training; STAIR, stair climbing-based high-intensity interval training.

Seven hundred sixty one records were screened, and 273 individuals were identified as eligible. Of those, twenty participants who met the eligibility criteria were enrolled in the study after providing written informed consent. Two participants voluntarily withdrew from the study due to time constraints relating to lack of availability for the physiological testing visits, leaving a total of 18 participants who completed the study, with *n* = 9 in each group. The flowchart of participants is depicted in Figure 2. Eighteen participants with CAD (61 ± 8 years) were included, with nine randomized to the TRAD group (61 ± 8 years; 8M/1W), and nine randomized to the STAIR group (62 ± 6 years; 8M/1W). Baseline characteristics of the participants can be found in Table 1. No adverse events were recorded through the duration of the study, and one participant, randomized to the STAIR group, was diagnosed with osteoarthritis of the ankle however was able to continue with their exercise protocol.

**Table 1:** Participant characteristics.

	**Baseline**	
	**TRAD (*n* = 9)**	**STAIR (*n* = 9)**
Sex (M/W)	(8/1)	(8/1)
Age (years)	61 ± 8	62 ± 6
Height (cm)	170 ± 12	175 ± 6
Body mass (kg)	89 ± 12	90 ± 11
BMI (kg/m^2^)	29.7 ± 4.1	29.8 ± 3.3
Resting SBP (mmHg)	116 ± 18	113 ± 17
Resting DBP (mmHg)	71 ± 10	78 ± 7
Resting HR (bpm)	68 ± 10	74 ± 12
**Clinical**		
STEMI (*n*, %)	1 (11.1)	2 (22.2)
NSTEMI (*n*, %)	5 (55.6)	5 (55.6)
Angina (*n*, %)	2 (22.2)	2 (22.2)
PCI (*n*, %)	5 (55.6)	7 (77.8)
CABG (*n*, %)	4 (44.4)	2 (22.2)
Time since event (weeks)	7.6 ± 4.2	8.6 ± 5.3
**Medications**		
Beta-blockers (*n*, %)	7 (77.8)	9 (100)
ACE inhibitors (*n*, %)	7 (77.8)	6 (66.6)
ASA (*n*, %)	9 (100)	9 (100)
Lipid lowering (*n*, %)	9 (100)	9 (100)
Metformin (*n*, %)	2 (22.2)	1 (11.1)
**CVD risk factors**		
Previous smoking history (*n*, %)	3 (33.3)	2 (22.2)
T2DM (*n*, %)	3 (33.3)	1 (11.1)
Hypertension (*n*, %)	8 (88.9)	6 (66.7)
Previous cardiac event (*n*, %)	5 (55.6)	0 (0)
Dyslipidemia (*n*, %)	8 (88.9)	6 (66.7)

*Data are mean ± SD. BMI, body mass index; BP, blood pressure; HR, heart rate; STEMI, ST-elevation myocardial infarction; NSTEMI, non-ST-elevation myocardial infarction; PCI, percutaneous intervention; CABG, coronary artery bypass graft; ACE, angiotensin-converting enzyme; ASA, acetylsalicylic acid; CVD, cardiovascular disease; T2DM, type 2 diabetes mellitus*.

**Figure 3:**
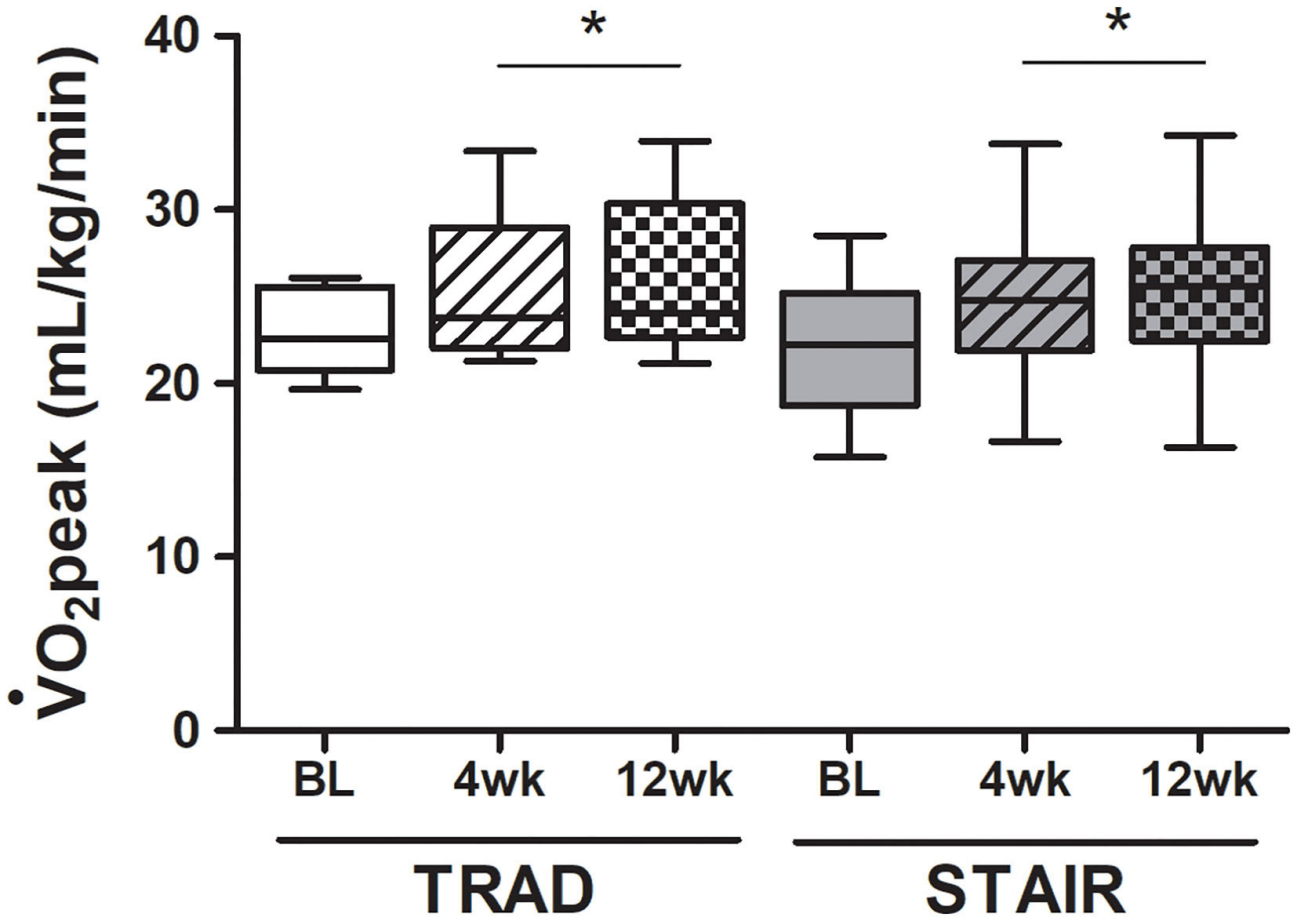
V°O_2_peak improvements following 4 weeks of supervised and 8 additional weeks of unsupervised exercise training of both TRAD and STAIR programs. Data is reported as a boxplot with the minimum, median and maximum values represented (*n* = 9/group). BL, Baseline; *significantly different from Baseline (*p* = 0.001), using a Two-way repeated measures ANOVA.

$\dot{\text{V}}{\text{O}}_{2\text{peak}}$ was higher after both 4 and 12 weeks of training vs. baseline (main effects), with no between-group differences (*p* = 0.994). Relative $\dot{\text{V}}{\text{O}}_{2\text{peak}}$ for TRAD was 22.9 ± 2.5, 25.3 ± 4.4, and 26.5 ± 4.8 mL/kg/min and STAIR was 21.4 ± 4.5, 23.4 ± 5.6, and 25 ± 6.2 mL/kg/min at baseline and after 4 and 12 weeks of training (Figure 3). $\dot{\text{V}}{\text{O}}_{2\text{peak}}$ was measured with the participant exercising on either an electronically braked cycle ergometer (*n* = 9; TRAD: *n* = 4, STAIR: *n* = 5) or a treadmill (*n* = 9; TRAD: *n* = 5, STAIR: *n* = 4), and was kept consistent for each individual for all measurement time points. All changes in cardiometabolic outcomes are reported in **Table 3**. Absolute $\dot{\text{V}}{\text{O}}_{2\text{peak}}$ improved over time (*p* = 0.001) with no differences between groups (*p* = 0.550).

Exercise protocol data for the supervised sessions can be found in Table 2. During supervised training the STAIR group attained a higher %HR_peak_ (*p* < 0.001), %HRR (*p* = 0.012), and peak training HR (*p* = 0.002) than the TRAD group. As expected, the TRAD group spent more time exercising at their prescribed exercise intensity compared to the STAIR group (*p* < 0.001). There were no differences in peak RPE between groups (*p* = 0.402).

**Table 2:** Average responses during supervised and unsupervised exercise training sessions.

	**TRAD (*n* = 9)**	**STAIR (*n* = 9)**	***P*-value**
**Supervised**			
Exercise intensity (%HR_peak_)	89 ± 1	106 ± 11^*^	≤ 0.001
Exercise intensity (%HRR)	67 ± 4	99 ± 9^*^	0.012
Peak training HR (bpm)	111 ± 13	131 ± 9^*^	0.002
Peak RPE	13 ± 2	12 ± 2	0.402
Total exercise time per session (min)	36.7 ± 1.1	7.1 ± 0.1^*^	0.009
**Unsupervised**			
HR_peak_/week (bpm)	111 ± 9	126 ± 13^*^	0.018
Exercise intensity (%HRR)	77 ± 6	109 ± 7^*^	0.004
Exercise intensity (%HR_peak_)	87 ± 8	96 ± 8	0.055
Total exercise time per session (min)	24.2 ± 17.0	10.0 ± 3.2^*^	0.036
% adherence	104 ± 75%	100 ± 105%	0.924

*Data are mean ± SD. TRAD, traditional moderate intensity continuous exercise; STAIR, stair climbing-based HIIT; HRR, heart rate recovery; HR, heart rate*.

* *Significantly different from TRAD (P ≤ 0.05)*.

Exercise protocol data for the unsupervised sessions (weeks 5–12 of training) can be found in Table 2. During unsupervised exercise training, the STAIR group did not attain a higher exercise intensity than the TRAD group when measured by %HR_peak_ (*p* = 0.055), however they attained a greater peak training HR (*p* = 0.018) and exercised at a higher %HRR than the TRAD group (*p* = 0.004). The TRAD group spent more time exercising at their prescribed exercise intensity compared to the STAIR group (*p* = 0.036. Overall exercise adherence was high and not different between groups (*p* = 0.924).

Participants in both the TRAD and STAIR groups decreased their body mass (*p* = 0.004) and BMI (*p* = 0.004) between baseline and 12 weeks of exercise training, with no difference between groups (*p* = 0.718). There were no differences across time or between groups in resting HR, resting BP, or HR_peak_, or peak systolic BP during the CPET. There were also no differences across time or between groups in fasted glucose and insulin, LDL, triglycerides, total cholesterol, and non-HDL cholesterol concentrations. HDL was increased following 12 weeks of both TRAD and STAIR exercise (*p* = 0.017), and total CHOL:HDL was higher in the TRAD group compared to the STAIR group (*p* = 0.011).

Accelerometer data were successfully recorded for most participants, with 16 out of 18 wearing the accelerometer for ~21–23 h/day for 7 days over 90% of the time (Table 3). There was no change in total activity (kcals/day) or total steps/day (*p* = 0.640) across the intervention or between groups (*p* = 0.755). There was an increase in METs/day (*p* = 0.043) and a decrease in sedentary time (*p* = 0.027) in both groups after 12 weeks of exercise training. There were no differences in light or moderate activity across the intervention or between groups, and no vigorous activity was recorded for any participant (Table 3).

**Table 3:** Outcomes measures after 4 weeks of 6 supervised sessions and 8 additional weeks of unsupervised exercise training for participants randomized into either traditional cardiac rehabilitation (TRAD, *n* = 9) or stair climbing-based high-intensity interval exercise (STAIR, *n* = 9).

	**TRAD**			**STAIR**					
	**Baseline**	**4 weeks**	**12 weeks**	**Baseline**	**4 weeks**	**12 weeks**	***P*-value (time)**	***P*-value (group)**	*P*-value (time^*^group)
Absolute $\dot{\text{V}}{\text{O}}_{2\text{peak}}$ (L/min)	2.1 ± 0.3	2.2 ± 0.4	2.3 ± 0.5^*^	1.9 ± 0.4	2.1 ± 0.5	2.2 ± 0.5^*^	0.001	0.550	0.994
HR_peak_ (bpm)	130 ± 18	132 ± 18	133 ± 17	124 ± 15	133 ± 16	124 ± 20	0.338	0.585	0.298
Body mass (Kg)	89 ± 12	88 ± 11	87 ± 11^*^	92 ± 11	90 ± 11	89 ± 12^*^	0.004	0.718	0.887
BMI (kg/m^2^)	29.7 ± 4.1	29.4 ± 3.6	29.0 ± 3.8^*^	29.8 ± 3.3	29.4 ± 3.1	29.1 ± 3.6^*^	0.004	0.975	0.928
Resting HR (bpm)	68 ± 10	69 ± 14	64 ± 12	71 ± 7	74 ± 12	69 ± 10	0.069	0.747	0.120
**Resting blood pressure (mmHg)**									
Systolic	116 ± 18	123 ± 26	120 ± 19	113 ± 17	117 ± 16	116 ± 11	0.395	0.660	0.866
Diastolic	71 ± 10	77 ± 11	72 ± 8	78 ± 7	75 ± 10	76 ± 6	0.212	0.283	0.097
	130 ± 18	132 ± 18	133 ± 17	124 ± 15	133 ± 16	124 ± 20	0.338	0.585	0.298
Fasted glucose (mmol/L)	5.2 ± 0.6	5.8 ± 1.4	5.7 ± 1.3	6.0 ± 1.1	5.8 ± 1.0	5.8 ± 0.9	0.927	0.138	0.314
Fasted insulin (pmol/L)	11.7 ± 6.2	14.9 ± 10.4	11.7 ± 9.7	8.1 ± 3.7	9.3 ± 4.7	9.6 ± 7.0	0.126	0.132	0.233
HDL (mmol/L)	0.86 ± 0.2	1.0 ± 0.2	1.0 ± 0.3^*^	1.2 ± 0.4	1.2 ± 0.4	1.3 ± 0.4^*^	0.017	0.208	0.836
LDL (mmol/L)	1.3 ± 0.4	1.5 ± 0.7	1.6 ± 0.5	1.4 ± 0.4	1.3 ± 0.4	1.3 ± 0.4	0.979	0.670	0.748
Triglycerides (mmol/L)	1.1 ± 0.4	1.4 ± 0.4	1.1 ± 0.3	0.86 ± 0.2	0.9 ± 0.1	0.8 ± 0.3	0.043	0.020	0.175
Cholesterol (mmol/L)	2.7 ± 0.6	3.1 ± 0.8	3.0 ± 0.7	3.0 ± 0.8	2.9 ± 0.5	3.0 ± 0.7	0.362	0.859	0.277
Non-HDL cholesterol (mmol/L)	1.9 ± 0.5	2.1 ± 0.7	2.1 ± 0.6	1.8 ± 0.5	1.7 ± 0.4	1.7 ± 0.5	0.674	0.348	0.368
Total CHOL:HDL ratio	3.2 ± 0.4	3.2 ± 0.5	3.1 ± 0.6	2.6 ± 0.5	2.6 ± 0.5	2.4 ± 0.4	0.155	0.011	0.858
**Accelerometer values**									
Wear time (h)	22 ± 0.9	22 ± 1.0	23 ± 1.0	24 ± 0.3	23 ± 1.5	22 ± 3.4	0.062	0.214	0.517
Wear %	94 ± 3	93 ± 4	93 ± 6	99 ± 1	97 ± 3	91 ± 15	0.138	0.495	0.301
Activity (kcals/day)	1,806 ± 485	1,953 ± 573	2,027 ± 446	1,744 ± 583	2,525 ± 1,953	1,879 ± 696	0.124	0.569	0.755
Steps/day	12,272 ± 2,367	12,603 ± 2,894	13,816 ± 3,055	10,520 ± 4,376	11,334 ± 4,898	13,739 ± 7,770	0.086	0.708	0.640
METs/day	1.5 ± 0.1^£^	1.6 ± 0.2	1.6 ± 0.2	1.5 ± 0.2^£^	1.6 ± 0.3	1.6 ± 0.3	0.043	0.771	0.816
Sedentary (min)	689 ± 110^£^	637 ± 120	629 ± 68	781 ± 164^£^	678 ± 141	656 ± 186	0.027	0.571	0.373
**MacNew quality of life**									
Emotional	74 ± 3	80 ± 2^*^	84 ± 2^*^	83 ± 3	83 ± 2	82 ± 2	0.033	0.243	0.011
Physical	65 ± 3	76 ± 2^*^	80 ± 3^*^	74 ± 3	77 ± 2^*^	79 ± 3^*^	0.001	0.922	0.104
Social	61 ± 4	76 ± 3^*^	81 ± 3^*^	74 ± 4	79 ± 3^*^	80 ± 3^*^	≤ 0.001	0.139	0.056
**IPAQ**, Total exercise (MET min/week)	2,468 ± 583	3,346 ± 786	5,273 ± 936	2,509 ± 618	3,133 ± 781	2,923 ± 993	0.080	0.203	0.312
**IPAQ**, Vigorous (MET min/week)	600 ± 295	1,609 ± 357^*^	2,978 ± 702^*^	760 ± 312	1,380 ± 378^*^	1,020 ± 744^*^	0.010	0.059	0.024
**IPAQ**, Moderate (MET min/week)	353 ± 315	760 ± 460	933 ± 386	908 ± 334	1,200 ± 488	548 ± 409	0.730	0.433	0.196
**IPAQ**, Walking (MET min/week)	1,514 ± 285	977 ± 256	1,362 ± 367	842 ± 302	553 ± 271	1,355 ± 389	0.088	0.533	0.254
**PACES**	–	109 ± 17	104 ± 21	–	101 ± 13	98 ± 15	0.376	0.388	0.903

*Data are mean ± SD. BMI, body mass index; HR, heart rate; LDL, low-density lipoprotein; HDL, high-density lipoprotein; CHOL, cholesterol; MET, metabolic equivalent; IPAQ, international physical activity questionnaire; MET, metabolic equivalent; PACES, physical activity enjoyment scale*.

* *Significantly different from Baseline P ≤ 0.05*.

£ *Significantly different from 12 weeks P ≤ 0.05*.

All participants improved their QLMI scores in both the physical and social categories, across the intervention (*p* < 0.001). The TRAD group improved in the emotional category from baseline to the 12 weeks (*p* = 0.033) with no change in the STAIR group (Table 3). Self-reported physical activity calculated as MET min/week from the IPAQ was not different across the intervention in either group (*p* = 0.312), although time spent in vigorous activity was increased after 4 weeks of supervised exercise and 8 weeks of unsupervised exercise when compared to baseline (*p* = 0.010), with the TRAD group reporting greater vigorous activity participation than the STAIR group [*p* (group × time) = 0.024]. There were no differences across time or between groups in time spent in moderate physical activity (*p* = 0.196) or time spent walking (*p* = 0.254). With respect to the maximum score of 126 arbitrary units, physical activity enjoyment (PACES) was rated high at baseline (TRAD: 109 ± 17 vs. STAIR: 101 ± 13) and was not different across time or between groups (Table 3) (*p* = 0.903).

## Discussion

Both the TRAD and STAIR protocols led to improved $\dot{\text{V}}{\text{O}}_{2\text{peak}}$ after just six supervised exercise sessions in the first 4 weeks of training that was maintained after an additional 8 weeks of unsupervised exercise training in our hybrid supervised and unsupervised cardiac rehabilitation setting in participants with stable CAD. These findings are noteworthy given the STAIR intervention involved much less time overall. Participants within both groups also showed improvements in body mass, BMI, and quality of life, and decreases in sedentary time.

Our primary outcome, and one of the most important clinical outcomes in cardiac rehabilitation, was a significant improvement in $\dot{\text{V}}{\text{O}}_{2\text{peak}}$. This improvement was observed early in the intervention, following six supervised exercise sessions, and was maintained after the 8 weeks of unsupervised exercise training. Both TRAD and STAIR groups showed improvements in $\dot{\text{V}}{\text{O}}_{2\text{peak}}$. The consensus in the literature is that HIIT induces similar (Currie et al., 2013b; Prado et al., 2016) if not superior (Moholdt et al., 2011; Villelabeitia Jaureguizar et al., 2016) improvements in $\dot{\text{V}}{\text{O}}_{2\text{peak}}$ vs. TRAD exercise training. Within our study, both TRAD and STAIR showed a ~3.5 mL/kg/min increase in $\dot{\text{V}}{\text{O}}_{2\text{peak}}$ or ~1 MET, which represents a clinically significant improvement, since 3.5 mL/kg/min higher $\dot{\text{V}}{\text{O}}_{2\text{peak}}$ was associated with ~15% reduction in risk of death (Keteyian et al., 2008). The STAIR group exercised for a shorter duration than the TRAD group (STAIR: 7.1 ± 0.1 vs. TRAD: 36.7 ± 1.1 min) and improvements in $\dot{\text{V}}{\text{O}}_{2\text{peak}}$, body mass and BMI were observed in both groups. Both groups reported a similar peak RPE during the exercise training, which was noteworthy as the prescribed peak exercise intensities were different, with some of the STAIR participants exercising at or above their CPET identified HR_peak_.

A novel and relevant aspect of this study was the combination of supervised and unsupervised exercise sessions. Previous research suggests that there is no difference between hospital- and home-based rehabilitative exercise in terms of major health outcomes, such as $\dot{\text{V}}{\text{O}}_{2\text{peak}}$ (Aamot et al., 2014; Anderson et al., 2017), and that highest adherence, and most improvement in health outcomes, were associated with programs which used a combined strategy of supervised and unsupervised programmes (Arrigo et al., 2008; Moholdt et al., 2011). Our $\dot{\text{V}}{\text{O}}_{2\text{peak}}$ results agree with the current literature, and adherence to the program, regardless of exercise protocol, was high. It seems, the combination of supervised and unsupervised exercise, along with the provision of an activity tracking device, proved to be effective for promoting program adherence regardless of protocol, and warrants further investigation.

Patients with stable CAD with greater levels of physical activity, quantified by self-report (Aamot et al., 2016) or objective assessment (Stewart et al., 2013; ter Hoeve et al., 2017), have a lower risk of mortality (Stewart et al., 2013). Regardless of measured physical activity (Prince et al., 2015) and decreased sedentary time, patients who are less sedentary and have a greater $\dot{\text{V}}{\text{O}}_{2\text{peak}}$ can still gain significant health benefits with cardiac rehabilitation (ter Hoeve et al., 2017). We report that both the TRAD and STAIR groups improved their METs/day and decreased their sedentary time, and their self-reported physical activity increased in terms of vigorous MET min/week across the intervention. These findings, in combination with our observations of improvements in $\dot{\text{V}}{\text{O}}_{2\text{peak}}$ are encouraging and suggest that both TRAD and HIIT are effective and lay groundwork for future larger trials.

Previous work, including our own, suggests that HIIT is safe, effective, and well-tolerated by cardiovascular disease patients (Currie et al., 2013a, 2015; Aamot et al., 2016; Wewege et al., 2018). A retrospective analysis of ~5000 patients with cardiovascular disease over 7 years of supervised cardiac rehabilitation exercise reported a low risk of acute cardiovascular events with HIIT and TRAD (Rognmo et al., 2012). The data presented here provides further evidence of the safety and effectiveness of HIIT, using a stair climbing-based intervention for outpatient cardiac rehabilitation. There were no adverse events with either TRAD or STAIR protocols, and overall adherence was high. Moreover, the increase in the emotional category of QLMI over time was likely a reflection of cardiac rehabilitation exercise in general and was not associated with any specific exercise training program. Furthermore, overall exercise enjoyment, for both groups, was good, with no differences between groups, whether supervised or unsupervised. Participants self-reported their exercise enjoyment, measured by the PACES questionnaire, for the TRAD group: 109 ± 17 and 104 ± 21, and for the STAIR group: 101 ± 13 and 98 ± 15 at the 4 and 12 week testing visits, respectively. Although standards for low and high enjoyment have not been established, a maximum score of 126 is the highest that can be achieved using the PACES questionnaire. Based on previous literature, physical activity enjoyment is related to perceived competence and personal preference, and in the present work, this could be associated with type and intensity of activity, or external factors, such as environmental conditions, competition, or preference for individual or group setting (Murrock et al., 2016).

### Limitations

The design of the existing cardiac rehabilitation program at our study site presented constraints but also increased the relevance of the study design. Their current standards typically include only four supervised exercise sessions prior to referral to a community-based program or facility and both treadmill and stationary cycle ergometers are typically used during CPETs. However, we added two more sessions to ensure adequate familiarization of our participants with the exercise sessions they would undertake during the unsupervised sessions. The addition of these two exercise sessions did not exceed the typical cardiac rehabilitation exercise programmes duration of 12 weeks (Lavie and Milani, 2006). The 8 weeks of unsupervised exercise may have limited the improvements seen in comparison to other studies of similar training duration, but using exclusively supervised exercise training (Rognmo et al., 2004; Currie et al., 2013a). We acknowledge that without a non-exercise training control group, it is not possible to determine with confidence if the observed increases in cardiovascular fitness are due to the exercise programs. Eligible participants for this study had been previously referred to cardiac rehabilitation exercise programmes as part of their post-cardiac event medical care and as such, it would have been unethical to subsequently randomize them to a non-exercising control group. Results of this study must be interpreted with caution owing to the small sample size and low statistical power. Despite our awareness of the sex bias in cardiac rehabilitation exercise programmes, we were only able to recruit two women for this study. The proportion of women was 11.1% of the total sample, compared to previously published work from a large Canadian database that suggests women represent 24.6% of the participants in cardiac rehabilitation exercise programmes (Murrock et al., 2016). Regardless, a sex bias in multiple stages of CAD care, from diagnosis to rehabilitation, needs to be addressed.

## Conclusions

Alternative forms of cardiac rehabilitation, such as the practical option of stair climbing-based HIIT, are important complements to the currently accepted forms of rehabilitative exercise following a cardiac event. The findings from this study highlight benefits of both stair climbing-based HIIT and TRAD cardiac rehabilitation exercise. We found stair climbing-based HIIT to be safe, effective, and enjoyable for patients with stable CAD enrolled in cardiac rehabilitation. Adherence to exercise following a cardiac event is a main consideration of cardiac rehabilitation. The capacity to offer a variety of time-efficient and effective exercise training options for those patients enrolled in cardiac rehabilitation is likely critical for increasing adherence and preventing secondary cardiovascular disease. We view HIIT based on stair climbing as an effective option compared to TRAD programs in increasing cardiorespiratory fitness.

## Data Availability Statement

The raw data supporting the conclusions of this article will be made available by the authors, without undue reservation.

## Ethics Statement

The studies involving human participants were reviewed and approved by Hamilton Integrated Research Ethics Board under Project #3301. The patients provided their written informed consent to participate in this study.

## Author Contributions

ED and MM developed the concept. SV, EL, MJ, MG, and SP were consulted to further enhance the study. ED, SV, SO, CM, EL, and JD were involved in subject recruitment and data collection. ED and SV completed the data analysis and completed all suggested revisions from the authors. ED, SV, and MM reviewed the data. ED wrote the first draft, and ED and SV developed the figures. All authors provided the feedback and direction in the revision stage, and provided the feedback on the entire review and final approval for submission.

## Conflict of Interest

The authors declare no professional relationships with companies or manufacturers who will benefit from the results of the present study. The results of the present study do not constitute endorsement by ACSM. The authors declare that the results of the study are presented clearly, honestly, and without fabrication, falsification, or inappropriate data manipulation.
